# Industrial Robustness: Understanding the Mechanism of Tolerance for the *Populus* Hydrolysate-Tolerant Mutant Strain of *Clostridium thermocellum*

**DOI:** 10.1371/journal.pone.0078829

**Published:** 2013-10-21

**Authors:** Jessica L. Linville, Miguel Rodriguez, Miriam Land, Mustafa H. Syed, Nancy L. Engle, Timothy J. Tschaplinski, Jonathan R. Mielenz, Chris D. Cox

**Affiliations:** 1 Department of Civil and Environmental Engineering, University of Tennessee, Knoxville, Tennessee, United States of America; 2 Bioenergy Science Center, Oak Ridge National Laboratory, Oak Ridge, Tennessee, United States of America; 3 Biosciences Division, Oak Ridge National Laboratory, Oak Ridge, Tennessee, United States of America; 4 Institute for a Secure and Sustainable Environment, University of Tennessee, Knoxville, Tennessee, United States of America; Virginia Commonwealth University, United States of America

## Abstract

**Background:**

An industrially robust microorganism that can efficiently degrade and convert lignocellulosic biomass into ethanol and next-generation fuels is required to economically produce future sustainable liquid transportation fuels. The anaerobic, thermophilic, cellulolytic bacterium *Clostridium thermocellum* is a candidate microorganism for such conversions but it, like many bacteria, is sensitive to potential toxic inhibitors developed in the liquid hydrolysate produced during biomass processing. Microbial processes leading to tolerance of these inhibitory compounds found in the pretreated biomass hydrolysate are likely complex and involve multiple genes.

**Methodology/Principal Findings:**

In this study, we developed a 17.5% v/v *Populus* hydrolysate tolerant mutant strain of *C. thermocellum* by directed evolution. The genome of the wild type strain, six intermediate population samples and seven single colony isolates were sequenced to elucidate the mechanism of tolerance. Analysis of the 224 putative mutations revealed 73 high confidence mutations. A longitudinal analysis of the intermediate population samples, a pan-genomic analysis of the isolates, and a hotspot analysis revealed 24 core genes common to all seven isolates and 8 hotspots. Genetic mutations were matched with the observed phenotype through comparison of RNA expression levels during fermentation by the wild type strain and mutant isolate 6 in various concentrations of *Populus* hydrolysate (0%, 10%, and 17.5% v/v).

**Conclusion/Significance:**

The findings suggest that there are multiple mutations responsible for the *Populus* hydrolysate tolerant phenotype resulting in several simultaneous mechanisms of action, including increases in cellular repair, and altered energy metabolism. To date, this study provides the most comprehensive elucidation of the mechanism of tolerance to a pretreated biomass hydrolysate by *C. thermocellum*. These findings make important contributions to the development of industrially robust strains of consolidated bioprocessing microorganisms.

## Introduction

Organic fuels, chemicals, and materials developed from plant biomass are among the leading options to meet sustainability requirements of the future [1]. Cellulosic biomass is an attractive feed stock for production of renewable fuels because of its widespread availability and relatively low cost [1]. The conversion process of cellulosic biomass to fuels utilizes biological fermentations. The most economic process combines the cellulase production, biomass hydrolysis, and sugar fermentation into a single step by a cellulose-fermenting microorganism in a consolidated bioprocessing (CBP) scheme [1,2]. CBP eliminates the cost of exogenous cellulase addition and consolidates capital equipment [2,3]. 

The plant cell walls of cellulosic biomass consist of several intertwined heterogeneous polymers, primarily cellulose, hemicelluloses (e.g., substituted xylans, mannans, ect.), pectin and lignin [4]. Various pretreatment methods are designed to make the sugars more available for subsequent hydrolysis and fermentation steps through the breakdown of the cell wall and the degradation of cellulose, hemicelluloses, and lignin matrix [4,5]. Most pretreatment processes produce compounds that are inhibitory to the organism used for fermentation. These inhibitory compounds come from the partial degradation of biomass components and include carboxylic acids (primarily acetic acid), furfural, 5-hydroxymethylfurfural (HMF), phenolic compounds, and inorganic salts [4]. The furan derivatives HMF and furfural are formed at high temperature and pressure as degradation products of hexoses and pentoses, respectively [4,6]. The phenolic compounds originate from the degradation of lignin [4,6]. Different biomass feedstocks and pretreatment methods produce different combinations of inhibitory compounds that stress the microorganisms [5-7]. Acetic acid, furfural and HMF are the most studied inhibitory compounds [4,5].

A variety of cellulolytic microorganisms are under development for biofuel production, but these organisms are typically poorly characterized [8,9]. An exception is *Clostridium thermocellum* which has been the subject of significant research for decades. *C. thermocellum* is a Gram-positive, anaerobic, thermophilic, cellulolytic bacterium that can rapidly solubilize biomass and use cellulose as a carbon and energy source [8,10,11]. High efficiency cellulose hydrolysis is aided by the cell surface attached multi-enzyme protein complex termed the cellulosome [11,12]. *C. thermocellum*’s cellulolytic ability gives it an advantage over organisms that are currently used for bioethanol production (e.g., yeast and *Zymomonas*), which can only ferment nonpolymeric carbohydrates, and has the potential to be a model organism for CBP [9,13]. *C. thermocellum* produces a number of industrially important fermentation products in addition to ethanol, including acetic acid, formic acid, lactic acid, and hydrogen[9]. Inhibitory compounds have been shown to reduce the rate of ethanol production and the overall yield [14,15]. Improved tolerance to inhibitory compounds found in pretreated biomass hydrolysate should improve the fermentation process and increase economic feasibility of consolidated bioprocessing. 

*C. thermocellum* 27405 is among the rapidly growing number of microorganisms whose genome has been sequenced and annotated, and serves as a baseline for comparison to strains improved through engineering or evolution. Thus genomic sequencing can assist in determining the possible mutations responsible for an evolved phenotype. When genome sequencing is applied in a longitudinal manner, in which the genome is sequenced at various time points during its evolution, the mutation rate can be determined. Studies have shown that the mutation rate increases in the later phase of the adaption due to the development of a mutator phenotype [16]. However, the genome sequence that is obtained represents a population average rather than the sequence of any individual bacterium [17]. Therefore, it is not possible to characterize a species from a single genome sequence [17]. The best approximation to describe a species uses the concept of the pan-genome from multiple single cell isolates [17]. The pan-genome can be divided into three elements: a core genome that is shared by all single cell isolates; a set of shared mutations that are shared by some but not all single cell isolates; and a set of isolate-specific mutations that are unique to each single cell isolate [17]. When the pan-genome is combined with population samples, a fourth category of discarded mutations can be identified which occur in the population samples but not in the single cell isolates. The pan-genome reflects the selective pressure to generate new adaptive combinations by recombining and constantly restructuring gene variants (alleles) in the population [17]. RNA Sequencing (RNA-seq) is an emerging technology that is being used for expression studies and it offers several advantages over DNA microarrays such as better detection of genes expressed at low levels [18]. RNA-seq analysis is particularly relevant for controlled experiments comparing expression in wild type and mutant strains of the same microorganism [19].

To date, the majority of genetic regulation studies for *C. thermocellum* have focused on the cellulosome [10,20-25] or ethanol tolerance [3,8,13,26]. Only a few studies have looked at gene regulation of *C. thermocellum* on a global level [9,11,27]. At this time, there is no known research that investigates the effects of hydrolysates from pretreated biomass on *C. thermocellum* gene expression. Current research suggests that comparing the change in phenotype between a wild type strain and a mutant strain of bacteria can give the greatest insight into the genes which are essential to the change in phenotype between the two strains. Therefore, in this study, we created a mutant population of *C. thermocellum* tolerant to 17.5% v/v *Populus* hydrolysate from which we isolated seven single cell colonies. We have sought to understand the mechanism of tolerance by (1) sequencing the genomes of the wild type strain (WT), intermediate population samples, and final isolate samples; (2) conducting comparative fermentative growth studies with the wild type and a final mutant isolate stain; and (3) comparing gene expression between the two strains during fermentative growth studies. 

## Material and Methods

### Strain and Culture Conditions

*Clostridium thermocellum* ATCC 27405 was obtained from Prof. Herb Strobel, University of Kentucky collection and denoted as the wild type strain (WT). In all experiments the cells were grown in media for thermophilic clostridia (MTC) with a substrate loading of 5 g/L cellobiose. The medium was composed of 0.336 g/L potassium chloride [KCl], 0.25 g/L ammonium chloride [NH_4_Cl], 1.00 g/L magnesium sulfate heptahydrate [MgSO_4_*7H_2_O], 1.70 g/L potassium phosphate [KH_2_PO_4_], 0.50 g/L MOPS [C_7_H_14_NO_4_S], 0.15 g/L calcium chloride dehydrate [CaCl_2_*2H_2_O], 1.75 g/L trisodium citrate dehydrate [Na_3_C_6_O_7_*2H_2_O], 0.6 g/L urea [CH_4_N_2_O], 1.00 g/L L-cysteine HCL, 0.30 mg/L resazurin, 2.0 mL of 1000x MTC minerals and 1.25 mL of 50x MTC vitamins [28,29]. All chemicals were reagent grade and obtained from Sigma (St. Louis, MO), unless otherwise indicated. The initial medium pH was approximately 6.8 - 7.0. The cells were grown in 25 mL Balch tubes (Bellco Glass, Inc., Vineland, NJ) and were incubated in the dark in an orbital shaker at 58 °C and 100 rpm. 

### *Populus* hydrolysate preparation and analysis

Milled *Populus trichocarpa* hydrolysate was pretreated at the National Renewable Energy Lab (NREL) using a 20% w/w solid loading and dilute concentrations of H_2_SO_4_ at temperatures of 165-195 °C [30]. Solids were removed by filtration. The *Populus* hydrolysate was adjusted to a pH of 7.0 using 50% w/w NaOH and filter-sterilized before being added to the MTC medium at various concentrations. HPLC analysis used a LaChrom Elite system (Hitachi High Technologies America, Inc. Pleasanton, CA) equipped with a refractive index detector (Model L-2490) and UV lamp (Model L-242OU). Metabolites were separated at a flow rate of 0.5 mL/min in 5 mM H_2_SO_4_ using an Aminex HPX-87H column (Bio-Rad Laboratories, Inc, Hercules, CA). GC-MS analysis used sorbitol (10 μl of a 1 mg/mL aqueous solution) added to 10 μl of sample as an internal standard to correct for differences in derivatization efficiency and changes in sample volume during heating.  The sample was dried in a nitrogen stream and then dissolved in 500 μL of silylation–grade acetonitrile, followed by the addition of 500 mL N-methyl-N-trimethylsilyltrifluoroacetamide (MSTFA) with 1% trimethylchlorosilane (TMCS) (Thermo Scientific, Bellefonte, PA), and then heated for 1 h at 70 °C to generate trimethylsilyl (TMS) derivatives [31].  After 2 days, 0.5 to 1-mL aliquots were injected into an Agilent Technologies Inc. (Santa Clara, CA) 5975C inert XL gas chromatograph-mass spectrometer (GCMS), fitted with an Rtx-5MS with Integra-guard (5% diphenyl/95% dimethyl polysiloxane) 30 m x 250 µm x 0.25 µm film thickness capillary column. The standard quadrupole GC-MS was operated in the electron impact (70 eV) ionization mode; gas (helium) flow is set at 1.2 mL per minute with the injection port configured in the splitless mode. The injection port, MS Source, and MS Quad temperatures are set to 250 °C, 230 °C, and 150 °C, respectively. The initial oven temperature is held at 50 °C for 2 min and is programmed to increase at 20 °C per min to 325 °C and held for another 11 min, before cycling back to the initial conditions.

### Adaptation of *C. thermocellum* to *Populus* hydrolysate

Adaption of *C. thermocellum* was performed by serial transfers using crimp-sealed 25 mL Balch tubes containing fresh anaerobic medium at 24-hour intervals with increasing concentration of *Populus* hydrolysate [32]. The tubes were sealed empty and purged with N_2_ and sterilized by autoclaving at 121 °C. The tubes were then injected with 9.5 mL MTC medium containing cellobiose and a known concentration of *Populus* hydrolysate. Each inoculation/transfer was 5% volume (0.5 mL). The optical density (OD_600_) was measured directly (without sampling) using a Spectronic 21D (Milton Roy, Ivyland, PA). The OD was referenced to an uninoculated sample of the *Populus* hydrolysate medium incubated with the samples. The OD was used to determine the increase in cell growth over time. A given concentration of *Populus* hydrolysate was maintained for each transfer until the OD reached that of the WT strain at 24 hours. When the benchmark OD was obtained at 24 hours, the culture was transferred to a higher *Populus* hydrolysate concentration. The final adaptation culture, after 117 transfers, is tolerant to 17.5% v/v *Populus* hydrolysate. Various viable culture samples were stabilized with 33% glycerol and stored at -80 °C during the course of the mutation process [3]. 

### Isolation of single colonies

Agar (Fisher Scientific, Pittsburg, PA, USA) solution (15 g/L) was prepared with 5 g/L cellobiose MTC medium. The agar-containing bottles were then transferred into an anaerobic chamber (Coy Laboratory Products, Grass Lake, MI, USA) where the following manipulation occurred. The final adaption culture was plated with serial dilutions of (100 μL, 10 μL, 1 μL, and 0.1 μL) on quad- Petri plates (BD Biosciences, Bedford, MA, USA) and mixed with the agar solution. The plates were allowed to sit for 30 min to solidify the agar and then incubated at 55 °C. Colonies were picked using a needle after 32-48 hours incubation. Seven petri plates were filled with the agar solution and let dry in the anaerobic chamber for 2-3 days. A picked colony was streaked onto the pre-poured plates and incubated for an additional 32-48 hours. A picked colony was then transferred into a microcentrifuge tube with 1 mL sterilized DI water, which was mixed and injected into a crimp-sealed Balch tube with 9.5 mL of cellobiose MTC medium. The tube was incubated at 58 °C and 100 rpm. After about 24 hours, stock culture was prepared with 33% glycerol and stored at -80 °C [3]. These cultures were named isolates 1-7. 

### Genome resequencing and analysis

Genomic DNA for the WT strain, six intermediate mutant populations, and seven 17.5% v/v *Populus* hydrolysate tolerant mutant isolates were extracted using a Qiagen DNeasy kit (Qiagen, Valencia, CA). The DNA samples were shipped in dry ice to the DOE Joint Genome Institute (JGI, Walnut Creek, CA). The samples were sequenced using JGI’s whole-genome shotgun sequencing method, which began with the creation of DNA libraries [33]. Sequencing was performed from both sides of the library insert, producing paired ends, typically resulting in approximately 10X depth coverage for the single colony isolates and 50X depth coverage for the intermediate populations. Sequenced reads were aligned using MAQ [34]. A report with Single Nucleotide Polymorphisms (SNPs) and statistical analysis was returned (NCBI study accession number: SRP024303). Further analysis was conducted on the mutations using the Genome Resequencing Toolkit available from BESC Knowledgebase [35]. The “Function change predictor” tool available from the toolkit was used to evaluate potential changes in the protein function, namely a loss or a gain of a protein family (Pfam) domain. This tool calculates the Pfam score of the non-synonymous amino acid substitution within a Pfam domain. The Pfam score is calculated from the Position Specific Scoring Matrix (PSSM) scores of the Pfam model, taking into consideration the position of the substitution and the amino acid in the mutant and in the reference sequence. The resulting Pfam score of the mutation can be positive, which indicates a potential gain of function, or negative, which points to a potential loss of the protein function. Detailed documentation of these tools is available at http://cricket.ornl.gov/html/download/resequencing/ResequencingToolkitDocumentation_16Dec2011.pdf [35-37]. Multiple sequence alignment was conducted on the top 100 Blastp hits using Clustalw. Amino acids were termed ‘very conserved’ upon visual inspection showing 100% agreement or ‘non-conserved’ if there was no conservation at that position. Genes with unknown function or hypothetical proteins where analyzed with BLAST to identify homologus genes with known functions. 

### PCR DNA sequencing

Genomic sequencing data was validated using PCR amplification of genomic DNA. PCR primers were designed for 28 mutations. The mutations selected for validation included 19 ‘high confident’ mutations and 9 ‘false positive’ mutations found in PM Isolates. PCR primers were designed with approximately 150-200 bp on either side of the mutation. Both the genomic DNA of PM Isolates and WT was PCR amplified using a GeneAmp PCR System 9700 Thermal Cycler (Life Technologies, Grand Island, NY). The DNA samples were concentrated using Qiagen PCR clean-up kit (Qiagen, Valencia, CA) and eluted into H_2_O. The samples were sequenced at the Molecular Biology Resource Facility (MBRF) at the University of Tennessee (Knoxville, TN) using the Sanger sequencing method. After further clean-up, the samples were loaded into a 48 capillary array Applied Biosystems 3730 Genetic Analyzer using ABI’s POP7 polymer and run using the default long read modules. The data files generated were analyzed using Applied Biosystems Sequencing Analysis Software vs. 3.7. The genetic sequences were compared to the reference genome of *C. thermocellum* ATCC 27405 using BLAST to confirm 100% homology for the WT sequences and the presence or absence of the suspected mutation in the PM Isolate sequences. 

### Fermentation

Batch fermentations were conducted in triplicate in 1.5 L Q-plus jacketed glass fermentors (Sartorius Stedim Biotech, Bohemia, NY) using a 1 L working volume of MTC medium at 58 °C and 300 rpm, with pH controlled to 7.0 using 3N NaOH. Fermentors containing only 5 g/L cellobiose were sparged with filtered 20% CO_2_/80% N_2_ gas mixture and vigorously agitated overnight, followed by addition of the remaining medium components and *Populus* hydrolysate. *Populus* hydrolysate was added to the fermentors at 0%, 10%, or 17.5% v/v concentration. The fermentors were then sparged for an additional 4 hours with a 20% CO_2_/80% N_2_ gas mixture. The inoculum was grown overnight in 125 mL serum bottles (11-13 hrs) in 5 g/L cellobiose, diluted to an OD_600_ of 0.200 + 0.013, and added as 10% v/v to the fermentors. Isolate 6 was chosen as the *Populus* mutant (PM) strain. Well-mixed 5 mL aliquots of culture were harvested at regular intervals. Cell growth was monitored based on an increase in the optical density. The OD_600_ of the culture was measured in triplicate by a Spectramax Plus 384 (Molecular Devices, Sunnyvale, CA). Metabolite analysis was performed using HPLC as previously described. Statistical analysis of the difference in growth parameters was conducted using an Analysis of Covariance [38] and was considered significant if the p-value was less than 0.05. 

### RNA isolation

Fermentation samples for RNA isolations were harvested by centrifugation of a ~30 mL culture in 50 mL conical tubes at 8000 rpm and 4 °C for 10-15 min. The solid pellet fraction containing cells was flash frozen in liquid nitrogen and stored at -80 °C until further use. Total RNA was extracted from the cell pellets as follows. Briefly, the frozen cell solution was thawed on ice and an equal volume of TRIzol (Invitrogen, Carlsbad, CA) was added. The cell solution was divided into ~1 mL aliquots and added to 2 mL tubes containing 1 mL of 0.1 mm glass beads (Biospec Products, Bartesville, OK) ashed at 250 °C overnight. Cells were lysed by rapid agitation of the tubes at 6500 rpm for 1 min in three 20s-ON/20s-OFF cycles using the Precellys® bead beater (Bertin Technologies, France). Subsequently, the cell lysate (~0.8 mL) in TRIzol was phase separated by addition of 200 μL chloroform and the RNA was precipitated by addition of 500 μL isopropanol. The precipitated RNA pellet was washed with 1 mL of 75% ethanol and resuspended in 100 μL of RNase-free water. Any contaminating DNA was digested in-solution with a DNase-I (Qiagen, Valencia, CA) treatment and the RNA sample was purified using the RNeasy mini kit (Qiagen, Valencia, CA) as per the manufacturer’s instructions. Methods for fermentation experiments through RNA isolation techniques were adapted from Raman et. al (2011) [27]. 

### RNA sequencing

The RNA samples were shipped in dry ice to DOE Joint Genome Institute (JGI, Walnut Creek, CA). JGI prepared an indexed, directional, amplified Illumina RNA_seq library for each sample using the RiboZero kit for rRNA removal [33]. Triplicate fermentation samples were pooled into a single channel and sequenced using 10 2x100 channels. The reads were mapped to the reference transcriptome. A report from JGI was returned with a correlation analysis between replicate samples and contained counts (raw and normalized to the gene length and total number of counts for the sample) for known gene models. Further analysis of the gene expression level was conducted using JMP Genomics 10. The raw count data was log-2 transformed and normalized by Upper Quartile Scaling [39]. An analysis of variance (ANOVA) test was conducted using a false discovery rate of 5% and the WT in 0% v/v *Populus* hydrolysate as the baseline. A gene was considered statistically significantly differentially expressed if the log-2 difference was greater than 1 and the -log_10_(p) was greater than 1.714. The differentially expressed genes were then clustered using hierarchal clustering. 

### Gas Analysis

Gas analysis was performed using triplicate sacrificial crimp-sealed 25 mL Balch tubes containing 5 mL of 5 g/L cellobiose- MTC medium with either 0% or 10% v/v *Populus* hydrolysate . WT and PM samples were grown using sacrificial techniques described above and collected at 3 hour intervals. The H_2_ and N_2_ gas analysis was performed using a Thermo Scientific Focus Gas Chromatography (GC) system (Asheville, NC) fitted with an HP Plot (molecular Sieve 5A) 30 m x 0.53 mm x 50 µm film thickness capillary column and helium as the carrier gas. Both the inlet and detector was held at 125 °C. The initial oven temperature is held at 30 °C for 4 min and is programmed to increase at 8 °C per min to 45 °C and held for 2 min, before cycling back to initial conditions. The CO_2_ and air analysis was performed using a Hewlett Packard Series II 5890 GC fitted with an HP Plot Q 15 m x 0.53 mm x 40 µm film thickness capillary column and helium as the carrier gas. The inlet and detector was held at 55 and 180 °C, respectively. The initial oven temperature is held at 50 °C for 1 min and is programmed to increase at 5 °C per min to 72 °C and held for 0.5 min, before cycling back to initial conditions. In both cases, 100 µL sample of gas was injected into the GC. H_2_ and CO_2_ concentrations were correlated using N_2_. Cell growth and metabolite analysis was performed using techniques previously described. 

## Results

### *Populus* hydrolysate analysis

The *Populus* hydrolysate used in these experiments was analyzed by HPLC and GCMS. Table 1 shows the average concentration of metabolites found in four samples of the hydrolysate taken over the course of the experiments. Analysis of the effects of various feedstocks and pretreatment combinations on the formation and the accumulation of potentially inhibitory degradation products in biomass hydrolysate have been investigated [40]. Additional literature discusses inhibition by products formed during biomass pretreatment [4,5,40-43]. Many of the non-carbohydrate compounds are believed to be inhibitory in nature [42,44-47]. 

**Table 1:** Results of four *Populus* hydrolysate samples metabolite analysis by HPLC and GC-MS.

**Sugar**	**g/L**	**aliphatic acids**	**mg/L**
Cellobiose	3.7 + 0.2	lactic acid	157 + 25
Glucose	19.8 + 1.9	maleic acid	25.0 + 11.3
Xylose	38.9 + 2.5	succinic acid	82.2 + 6.4
galactose	2.7 + 0.3	fumaric acid	56.1 + 12.7
Arabinose	1.6 + 0.2	levulinic acid	1349 + 191
Mannose	5.7 + 0.4	itaconic acid	11.7 + 0.7
**aromatic acids**	**mg/L**	adipic acid	0.3 + 0.7
2-furoic acid	41.8 + 4.2	acetic acid	11150 + 1881
3,4-dihydroxybenzoic acid	43.9 + 9.2	arabinonic acid	49.9 + 6.0
salicylic acid	1.5 + 1.1	xylonic acid	772 + 202
syringic acid	84.9 + 29.1	**aldehydes and ketones**	**mg/L**
4-hydroxybenzoic acid	9567 + 1223	5-hydroxymethylfurfural	460 + 23
benzoic acid	44.1 + 3.6	Furfural	1198 + 165
hydroquinone	4.1 + 0.5	4-hydroxybenzaldehyde	0.9 + 0.3
catechol	1.0 + 0.1	syringaldehyde	146 + 54
glucuronic acid	981 + 270	vanillin	143 + 39
galacturonic acid	7965 + 1769		

The samples were taken over the time course of the experiments.

### Mutant development

**Figure 1:**
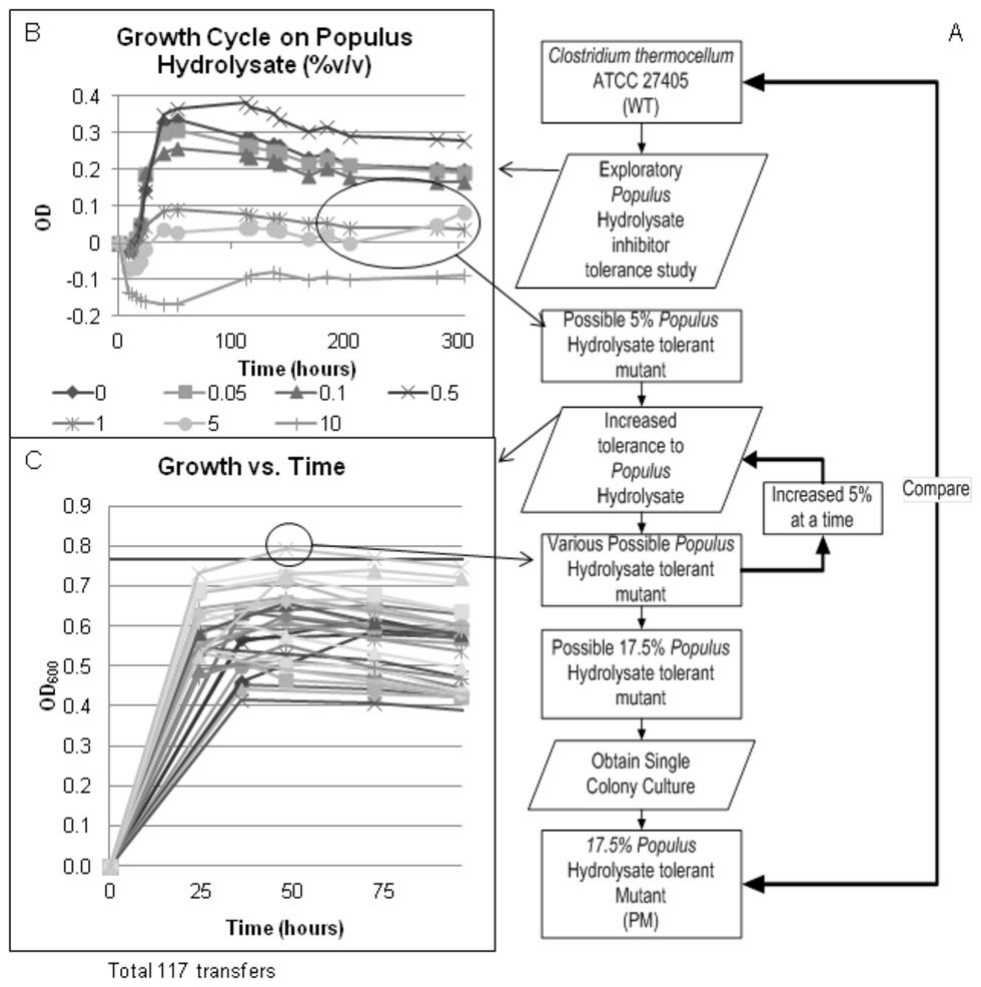
Flow chart for mutant development. (A) The flow chart shows the mutant development process from the initial WT strain through the final mutant. Single colony isolate 6 was chosen as the final 17.5% v/v *Populus* hydrolysate tolerant mutant strain (PM). (B) Initial growth of WT culture on various concentrations of *Populus* hydrolysate to determine inhibitory effect. The culture in 5% v/v *Populus* hydrolysate started growing late in the fermentation. (C) An example of serial transfers showing increased growth over time in 10% v/v *Populus* hydrolysate. Illustrative curves show each daily serial transfer.

The mutant development process can be seen in Figure 1, with the mutant development steps listed in Figure 1A. In order to determine the inhibitory effect of the *Populus* hydrolysate, the wild type strain (WT) of *C. thermocellum* was cultured with low concentrations of *Populus* hydrolysate (0.05% to 10% v/v *Populus* hydrolysate) in Figure 1B. During these early selection experiments the medium was prepared with only 1/3 of the vitamin solution compared to later experiments which explains the decreased growth of the WT in Figure 1B. Late in the fermentation (approximately 200 hours) the culture with 5% v/v *Populus* hydrolysate started to grow (Figure 1B). When cultured in fresh medium containing 5% v/v *Populus* hydrolysate this culture grew as well as the WT strain in standard medium (without hydrolysate) and was denoted as the final 5% v/v *Populus* hydrolysate tolerant mutant strain of *C. thermocellum*. Selection of a resistant culture began using 10% v/v *Populus* hydrolysate. The growth rate of each serial dilution was tracked at 24 hour intervals for 96 hours and plotted as optical density (OD) versus time (Figure 1C). The WT when grown in standard medium reaches a maximum OD_600_ of 0.750 within 24 hours; therefore, an OD of 0.750 was selected as the benchmark for a resistant culture. The OD of the culture in 10% v/v *Populus* hydrolysate at 24 hours slowly increased with each serial dilution transfer until the OD reached the required benchmark (Figure 1C). The concentration of *Populus* hydrolysate was then increased by 5% v/v and the process repeated. The benchmark was not reached after 30 serial dilutions when the culture was grown in 20% v/v *Populus* hydrolysate. At that point, the concentration of hydrolysate was reduced to 17.5% v/v *Populus* hydrolysate. This subculture was able to reach the benchmark OD and was denoted as the final 17.5% v/v *Populus* hydrolysate culture. As described, culture samples were saved throughout the process. After a total of 117 transfers, the 5% v/v *Populus* hydrolysate tolerant population generated the final 17.5% v/v *Populus* hydrolysate tolerant mutants. Seven single colony isolates were obtained from the final 17.5% v/v *Populus* hydrolysate tolerant mutant population. Isolates 1 and 4 grew poorly compared to the other isolates (Figure S1). Isolate 6 grew most similarly to the population average; therefore, it was selected for further strain comparisons and denoted PM for “*Populus* Mutant” (Figure 1A). The PM has a similar growth rate on insoluble cellulose (Avicel) compared to the WT strain despite being selected with cellobiose as the only carbon source (Figure S2). 

### Genetic changes during the mutation process

To determine the genetic basis of the *C. thermocellum* Populus hydrolysate- tolerant phenotype, the genomes of the WT strain, six intermediate populations and the seven single colony isolates were resequenced by JGI using the whole-genome shotgun sequencing method. JGI reported 224 putative mutations across all 14 samples (File S1) [33]. There were 20 putative differences compared to the reference genome in all of the sample sequences including the WT which were removed from consideration. The remaining 204 putative differences were screened for ‘high-confidence differences’ by removing mutations listed as false positive (fp), Illumina Sequence-specific error leading to a false positive (ISE), and structural variants (SV), resulting in 73 high confidence differences. 

In order to verify the JGI high throughput sequencing results, 19 of the high confidence mutations and 9 of the false positive mutations found in the PM Isolates were selected for verification by Sanger sequencing of PCR products (Table S1). Primers were designed to yield PCR products containing approximately 200 bp on either side of the mutation in question. PCR products were also generated for the WT as a negative control. The 19 high confidence mutations were selected randomly from PM Isolate 6. In each case the mutations identified by high throughput sequencing were identified in the PM sequences and the WT sequences resulted in 100% homology to the reference genome. Of the 131 false positive mutations identified by JGI, 62 were related to the presence of multiple long sequences of similar content in the *C. thermocellum* reference genome. Of the 69 potential false positive mutations that could be uniquely associated with a single sequence in the *C. thermocellum* reference genome via BLAST, only 11 appeared in PM isolates. PCR products were successfully obtained for 9 of these 11 potential mutations and in each case, both the WT and the PM sequences resulted in 100% homology to the reference genome, thereby confirming that no mutation occurred. Collectively these results suggest that the high throughput sequencing yields results that are reliable and that the false positive mutations identified by JGI can be safely ignored.

A longitudinal analysis was conducted on the six intermediate populations to determine when in the evolutionary process the mutations occurred. The number of mutations increased at a rate of 5+3 mutations between samples (Figure S3). The greatest increase in the number of mutations, 9 differences, occurred between the final 15% v/v *Populus* hydrolysate tolerant population and the final 17.5% v/v *Populus* hydrolysate tolerant population samples. The second highest increase in differences, 8 differences, occurred between the WT and the final 5% v/v *Populus* hydrolysate tolerant population samples. Although, there was no difference in the total number of mutations between the final 10% v/v *Populus* hydrolysate tolerant population and the intermediate 15% v/v *Populus* hydrolysate tolerant population sample, the actual mutations in these two populations were different from one another. 

**Figure 2:**
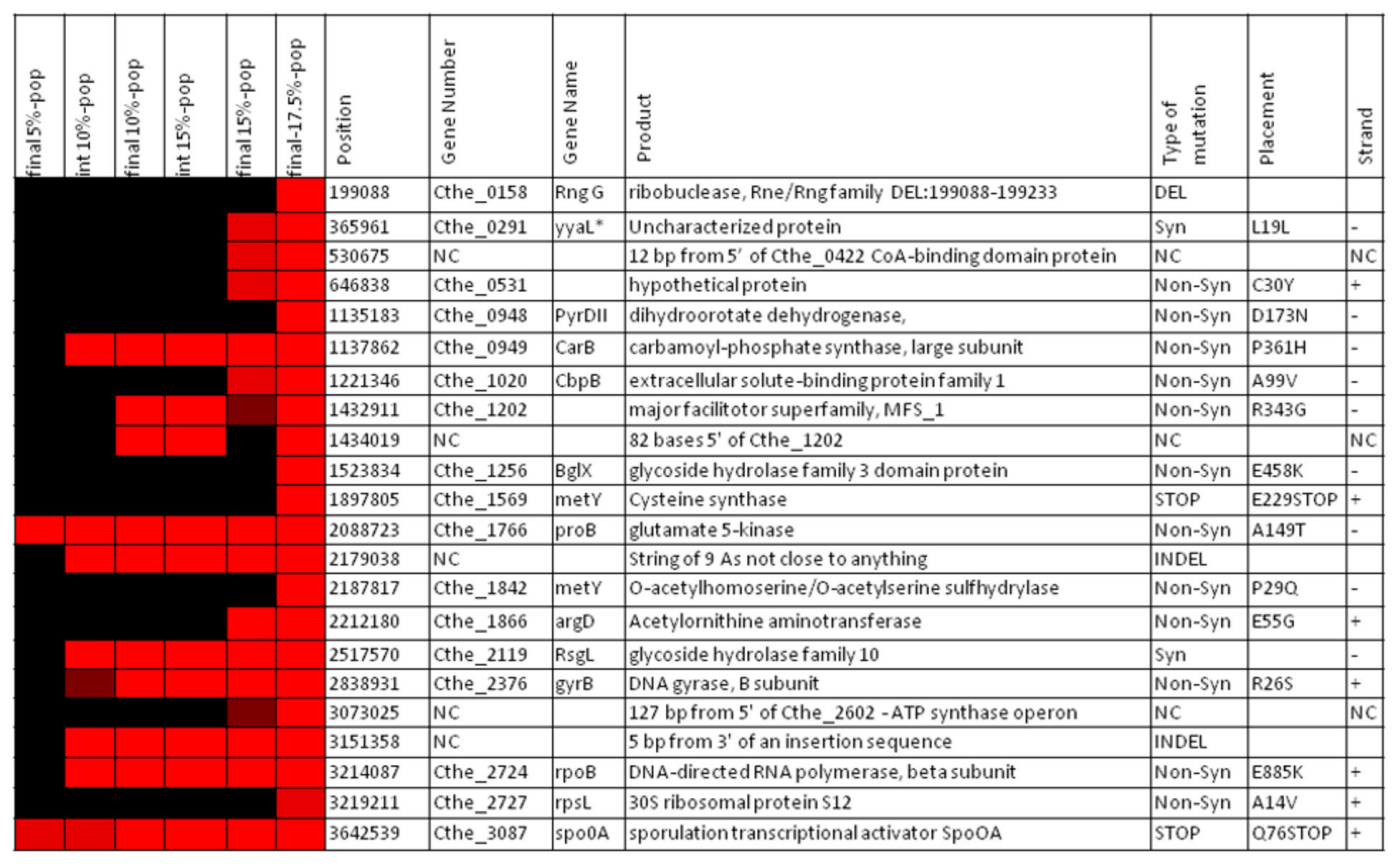
Core mutations common to all 7 single colony isolates. *Blast search of the uncharacterized protein returned yyaL as a homologus protein. Type of mutations: Syn: synonymous SNP, Non-Syn: Non-Synonymous SNP, NC: non-coding region, STOP: stop codon inserted, INDEL: insertion or deletion.

A pan genomic analysis (Figure S4) was conducted on the seven single colony isolates to determine the distribution of differences among the isolates. Each of the isolates contained an average of 27+2 differences. Of the 73 differences, 33% of the differences were found in the population samples but not in the isolate samples and were considered discarded mutations. Of the remaining differences, 32% were unique to one of the isolates, 5% were shared by multiple isolates, and 30% belonged to the core genome which is common to all of the isolates (designated as core mutations). Figure 2 lists the core mutations plus their annotated function, and when they occurred in the evolutionary process. 

Table S2 lists the unique and shared mutations for the seven single colony isolates. There are a total of 27 unique and shared mutations among the isolates. Only four of these mutations were detected in the final 17.5% v/v *Populus* hydrolysate tolerant mutant population sample at a concentration of < 2% of the population. None of the mutations were detected in earlier population samples. These mutations may explain some of the variability seen in the growth of the different single colony isolates (Figure S1). The two poor growing isolates have three mutations shared only by those two isolates: a synonymous SNP in a ribosomal protein (Cthe_0992), a non-synonymous SNP in an integrase family protein (Cthe_1127), and an insertion of 7bp near the 3’ end of an *AbrB* family transcriptional regulator (Cthe_2655). The two isolates only differ in two mutations. Isolate 1 has an INDEL in an acetate kinase (Cthe_1028) and Isolate 4 has a non-synonymous SNP in extracellular solute-binding protein (Cthe_1020). However, only the six unique and shared mutations contained in Isolate 6 will be considered further since the fermentation and RNA-seq experiments used this isolate. 

There was a nonrandom distribution of mutations with only 11% of mutations occurring in the non-coding region. The number of insertions or deletions (INDELs), synonymous single nucleotide polymorphous (SNPs), non-synonymous SNPs and STOP codons in the coding parts of the genome is presented in Figure S5. SNPs are the dominant type of mutation that occurred, and non-synonymous substitutions were approximately four times as abundant as synonymous. JGI did not report any transposon-mediated mutations. 

Further analysis of the distribution of mutations revealed an increase number of mutations at several locations termed hotpots. In order to be considered a hotspot, multiple mutations had to be located within a gene, in an operon, or along the same pathway and at least one of the genes had to be located in the core mutation genome (Figure 2). A manual analysis of the distribution of the mutations identified 8 putative hot spots (Table 2). Genes linked to the hot spots included 24 out of the 73 putative mutations and 14 of the 22 core mutations. 

**Table 2:** Mutational Hot spots identified in the sequenced genome.

**Hot Spot ID**	**Number of Mutations**	**Genes (# mutations, location**)	**Products**
1	5	**Cthe_0948 (1, CDS**)**, Cthe_0949 (1, CDS**)**, Cthe_1766 (1, CDS**)**, Cthe_1866 (1, CDS**), Cthe_2529 (1, CDS)	Production of various amino acids
2	5	**Cthe_2602 (2, NC**)*, Cthe_2603 (2, CDS), Cthe_2607 (1, CDS)	Single transcription unit Cthe_2602-9: Subunits for F-type H+-transporting ATPase
3	4	**Cthe_0422 (one NC and 1 CDS**) *, Cthe_1028 (1 CDS), Cthe_1029 (1 CDS)	Redox-sensing transcriptional repressor Rex and acetate formation from acetyl-CoA I
4	2	**Cthe_1569 (1 CDS**)**, Cthe_1842 (1 CDS**)	Homocysteine biosynthesis
5	2	**Cthe_1202 (one NC and 1 CDS**)	Major facilitotor superfamily, MFS_1
6	2	Cthe_1020 (2 CDS) *	Extracellular solute-binding protein family 1, PBP2_LTTR_substrate superfamily
7	2	**Cthe_3087 (2 CDS**) *	Sporulation transcriptional activator Spo0A
8	2	**Cthe_1256 (1 CDS**)**, Cthe_2119 (1 CDS**)	glycoside hydrolases

CDS, coding region, NC, non-coding region, bold genes belong to the core mutational genome, *only one of the mutations are in the core mutational genome

### Genes and mutations with unknown function

There are four mutations in the core genome with unknown function. Two of the mutations are INDELS located in non-coding regions and are located at positions 2179038 and 3151358. The two other mutations are in Cthe_0291 an uncharacterized protein, and Cthe_0531 a hypothetical protein. In an attempt to determine functionality of these genes a BLAST similarity search was run between the unknown genes and genes from public databases. The BLAST search for Cthe_0531 returned only genes with hypothetical protein annotations. However, the BLAST search for Cthe_0291 returned a number of genes annotated as the thioredoxin, yyaL, protein. 

### Fermentative Growth

**Figure 3:**
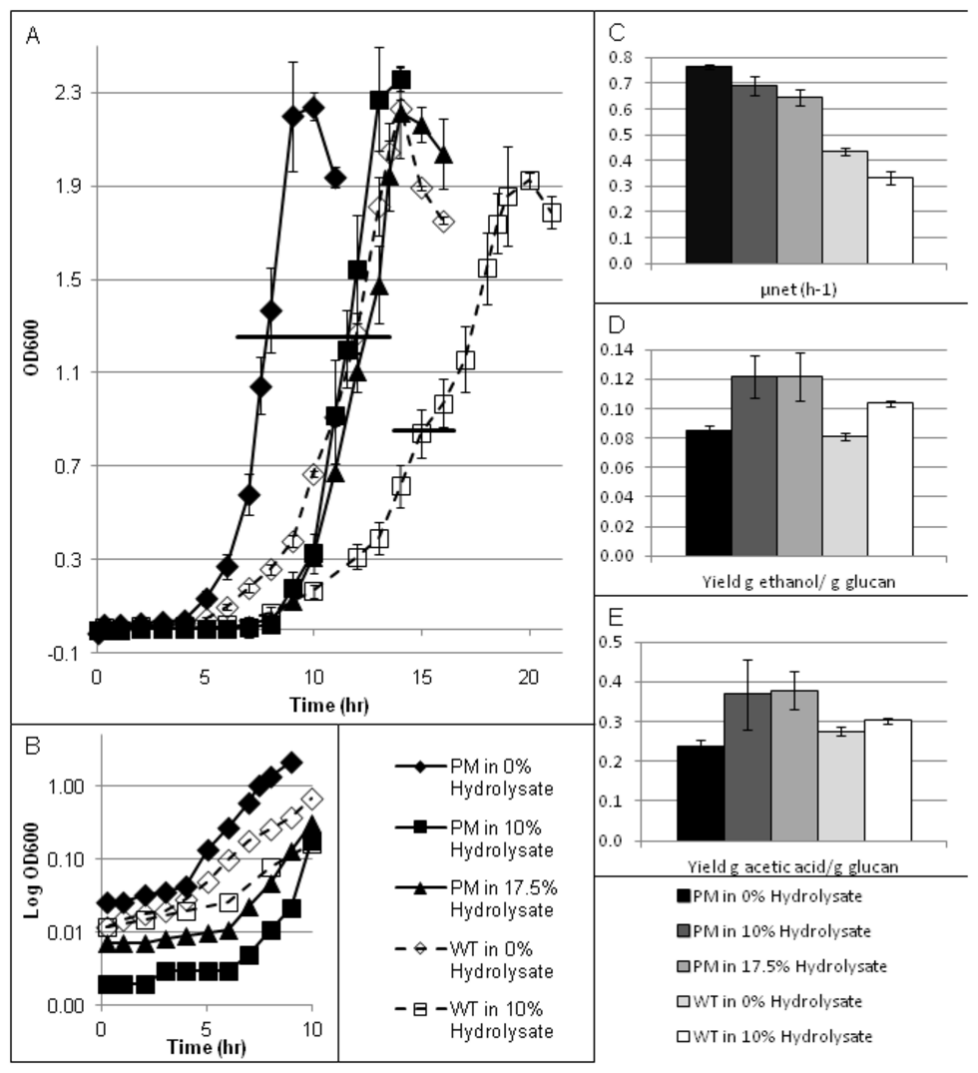
Growth comparison for WT and PM in various concentrations of *Populus* hydrolysate. (A) Optical density versus time. The horizontal bars indicate sample point for RNA-seq analysis, (B) the average OD on a log scale for the initial stages to emphasize the initiation of exponential growth, (C) net growth rate (ln(biomass/time, μ_net_), (D) yield of ethanol produced (g/L)/glucan utilized (g/L), and (E) Yield of acetic acid produced (g/L)/ glucan utilized (g/L).

Batch fermentations were conducted in triplicate in Q-plus fermentors to further evaluate the performance of the PM versus WT *C. thermocellum* strains in different levels of hydrolysate. The PM was grown in three test conditions: standard medium (0% v/v *Populus* hydrolysate), 10% v/v and 17.5% v/v *Populus* hydrolysate. The WT was grown in two test conditions: standard medium and 10% v/v *Populus* hydrolysate. The WT grown in the presence of 17.5% v/v *Populus* hydrolysate severely inhibited growth in an unpredictable manner (data not shown). Samples were taken at regular intervals from each fermentation unit based on their growth rate. Samples were analyzed for OD and metabolite concentration by HPLC. Figure 3 shows the average OD over the entire fermentation (Figure 3A) and the average OD on a log scale for the initial stages to emphasize the initiation of exponential growth (Figure 3B). The lag period lasted approximately 4 hours for the standard medium conditions and 6 hours for the hydrolysate medium condition for both the PM and WT strains. Figure 3 also shows the net growth rate (ln(biomass/time, μ_net_) (Figure 3C), ethanol yield (Figure 3D) and acetic acid yield (Figure 3E) for each of the test conditions over the exponential growth phase. Table S3 contains p-values indicating statistically significant differences in pair-wise comparisons of growth rates and product yields among the various strains and growth conditions. The net growth rate is calculated from the end of the lag phase until the time of maximum cell density. Less than 15% of the initial glucan concentration and less than 5% initial cellobiose remained at the end of the fermentations conducted in 0% and 10% v/v *Populus* hydrolysate. Approximately 19% and 8% of the initial glucan and cellobiose remained at the end of the PM fermentation with 17.5% v/v *Populus* hydrolysate. End products yields are calculated as a function of the amount of glucan utilized from the fermentation broth since the hydrolysate contained additional cellobiose and glucose. In brief, the PM had an increased growth rate when compared to the WT in the same condition. The PM was characterized by similar growth rates in 0% and 10% v/v *Populus* hydrolysate media, but the growth rate decreased in 17.5% v/v *Populus* hydrolysate medium. The growth rate of the WT decreased in 10% v/v *Populus* hydrolysate medium in comparison to 0% v/v *Populus* hydrolysate medium. The PM was characterized by a greater ethanol yield compared to the WT under the same test conditions. Ethanol yields also increased for both strains in the presence of *Populus* hydrolysate. Acetic acid yield were similar for both strains when grown under the same growth conditions. Acetic acid yield increased in the PM strain in the presence of hydrolysate, but remained constant for the WT strain. The p-value of the regression analysis can be seen in Table S3. 

### RNA-seq Analysis

**Figure 4:**
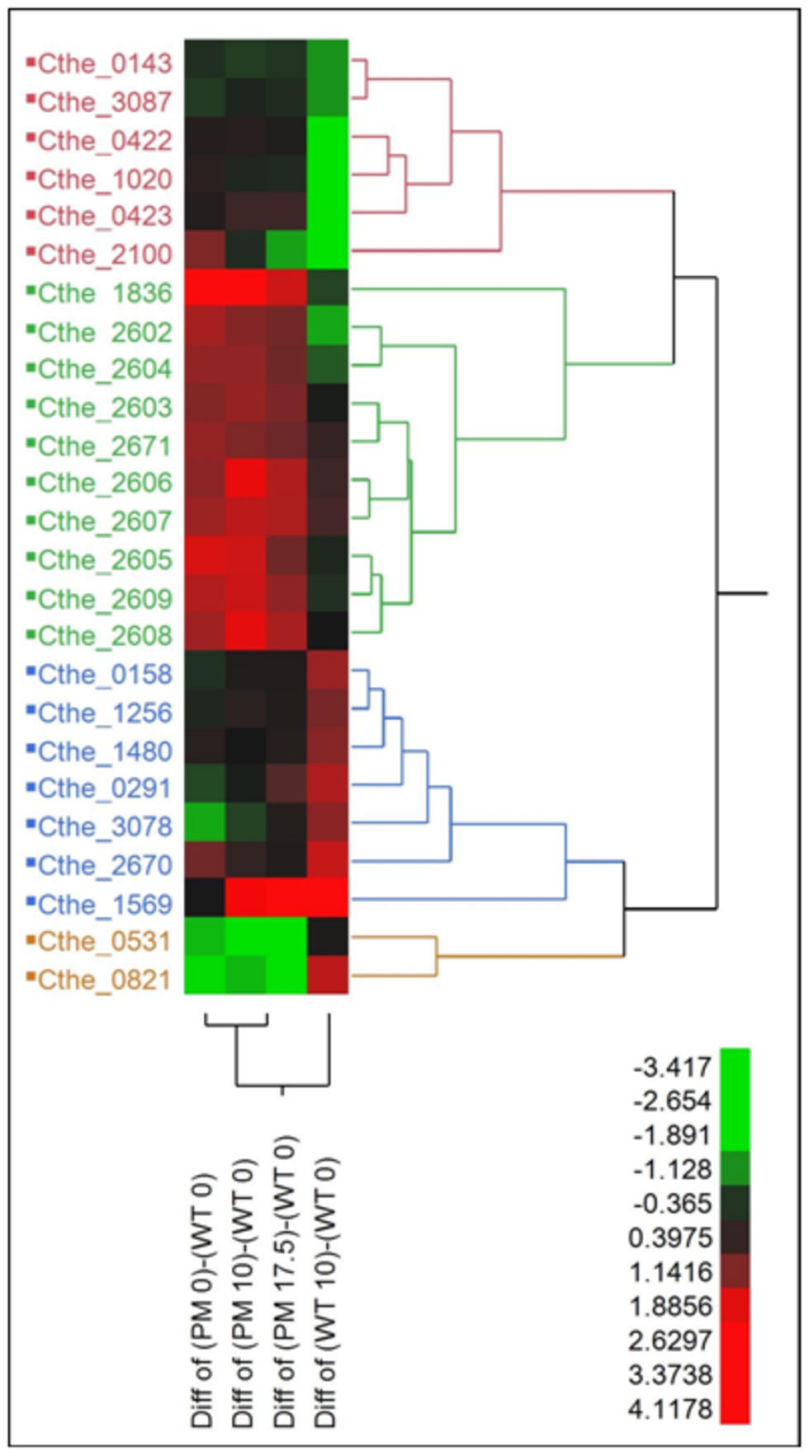
Hierarchical clustering of differentially expressed genes. The scale represents the log-2 transformed difference between the two conditions; therefore a change greater than one represents a >2-fold change in gene expression.

The samples for RNA analysis were harvested from the fermentors during the mid-log phase based on OD (see Figure 3A). Resulting RNA-seq data was used to elucidate the role of the genomic changes that helped confer the hydrolysate tolerant phenotype. Gene expression data were analyzed for genes that: (1) were mutated, (2) were located downstream or in an operon with a mutated gene, (3) were another copy of a mutated gene, or (4) were under regulatory control of a mutated gene. An ANOVA analysis was conducted on the resulting subset of 41 genes using a false discovery rate of 5% and the expression of the WT in 0% v/v *Populus* hydrolysate as the baseline. Of the 41 genes 25 of them were differentially expressed in at least one of the comparisons. The 25 differentially expressed genes were clustered using hierarchal clustering as shown in Figure 4. The differential expression profiles for the subset of 41 genes can be found in File S2. 

### Gas Analysis

Sacrificial fermentations were conducted in triplicate in 25 mL Balch tubes to evaluate the CO_2_ and H_2_ production of the PM versus WT *C. thermocellum* strains in 0% and 10% v/v *Populus* hydrolysate. Samples were taken at 3 hour intervals and analyzed for OD, metabolite concentration by HPLC, and gas concentration by GC. The growth rates in the Balch tubes were shown to be statistically equivalent to the growth rates in the Q-plus fermenters (Table S3) facilitating comparisons across the two reactor types. The production of CO_2_ or H_2_ versus glucan utilization for the WT and PM can be seen in Figure S6 part A and D respectively. Table S3 indicated that both strains had similar H_2_ production rates under the same experimental conditions. Statistical analysis shows that the PM produces slightly more CO_2_ than the WT in 0% v/v *Populus* hydrolysate medium, but similar amounts in 10% v/v *Populus* hydrolysate medium. 

## Discussion

Further analysis was conducted on the core mutations (Figure 2) with insight from the hot spot analysis (Table 2). The mutations were broken down into three basic categories: mutations beneficial to the tolerant phenotype, extraneous or detrimental mutations, and mutations of unknown function. 

### Mutations beneficial to the tolerant phenotype

**Figure 5:**
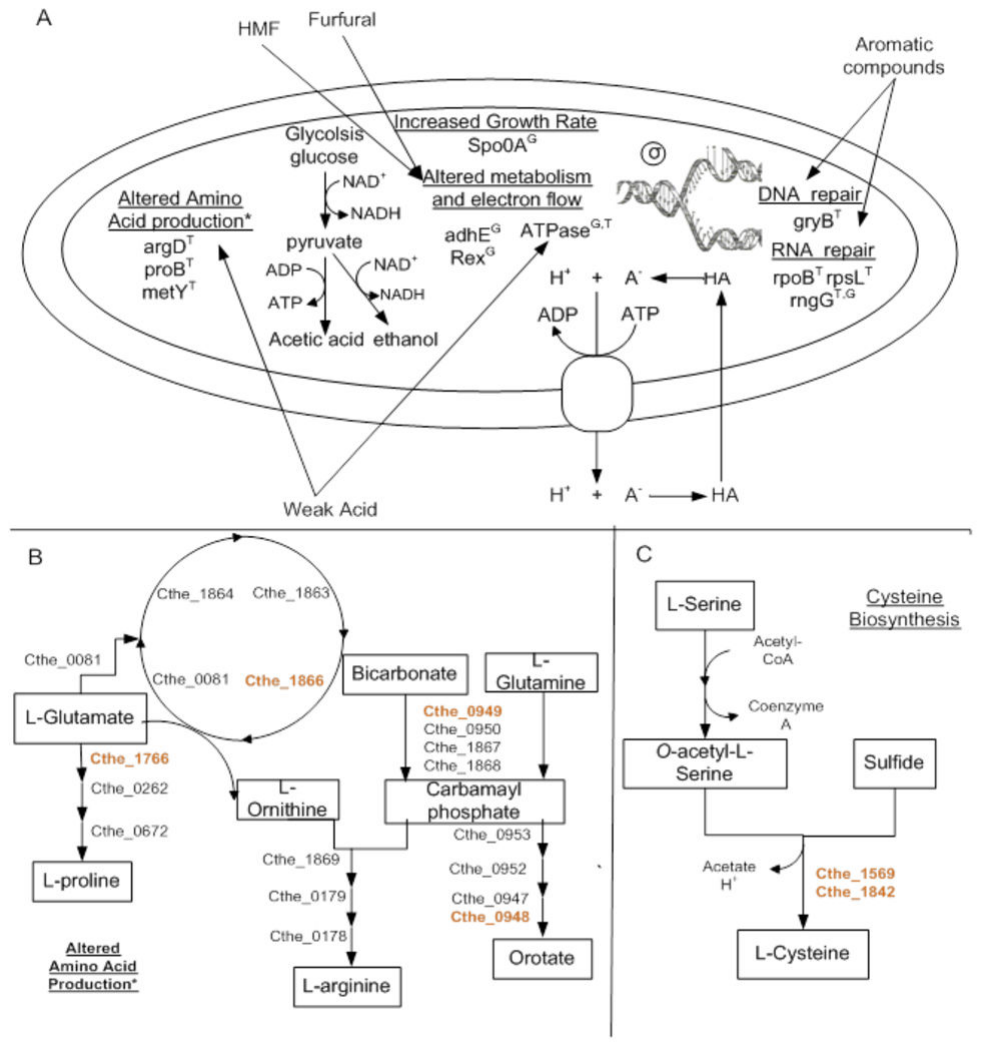
Mechanisms of tolerance. (A) Graphical representation for the mechanism of tolerance in PM. The superscript T means that the mutation is most likely is involved with enhanced tolerance by the mutatnt strain and the superscript G means that the mutation is most likely involve with enhanced growth rate of the mutatn strain (B) In depth look at the alter amino acid production (C) and in homocysteine biosynthesis (Taken in part from [55,56]).

Inhibitory compounds may affect the cell by damaging and denaturing biological molecules resulting in adverse outcomes including the improper folding of proteins, DNA damage, improper RNA unfolding and degradation, and the impairment of biophysical changes to cell membranes necessary for energy generation and the proper functioning of molecular pumps [6,48]. Figure 5 provides an overall schematic of the mechanisms of tolerance for the PM. The tolerant phenotype is the result of several simultaneous mechanisms of action, including increases in cellular repair, and altered energy metabolism. These and related mechanisms are apparently independent from each other and can involve genes or gene clusters widely dispersed on the chromosome [6]. The ‘tolerant’ phenotype can be broken down into two distinct changes in PM compared to the WT. The first is the PM’s enhanced growth phenotype which can be seen by the comparison of the growth rate of the two strains in 0% v/v *Populus* hydrolysate. The second is the PM’s enhanced tolerance to hydrolysis components which can be seen by the difference in the response to hydrolysate of the two strains (Figure 3A). 

### DNA repair and mRNA and protein turnover

Cells contain multiple lines of defense against protein aggregation, DNA and RNA damage, and membrane and organelle damage [6]. The PM has one mutation affecting a gene encoding for a protein related to DNA repair (Cthe_2376) and three mutations affecting genes related to transcription (Cthe_2724), translation (Cthe_2727) and RNA degradation (Cthe_0158). The mutations in Cthe_2376, Cthe_2724 and Cthe_2727 are all non-synonymous SNPS and Cthe_0158 has a deletion near the 3’ end. 

The *gyrB* gene (Cthe_2376) encodes an ATP-dependent DNA gyrase enzyme which cleaves and reseals double stranded DNA thereby introducing negative supercoils into DNA [49]. This activity is essential for DNA replication, transcription and recombination [49]. The non-synonymous SNP mutation in *gyrB* (R26S) occurs in a very conserved amino acid based on the Clustlw alignment (File S3). *C. thermocellum* contains a second copy of the *gyrB* gene (Cthe_0305) which is expressed at approximately one tenth of the level of Cthe_2376 in the WT under standard conditions. Neither copy of the *gyrB* gene is differentially expressed across the different conditions (data not shown). It is likely that Cthe_2376 is the dominant copy of the *gyrB* gene in *C. thermocellum*. Although, the potential change in specificity, selectivity or stability resulting from this mutation is unknown, the mutation may be beneficial for hydrolysate tolerance by reducing DNA replication errors.

The *rpoB* gene (Cthe_2724) encodes for the β-subunit of RNA polymerase. The RNA polymerase controls the flow of information from genotype to phenotype and is composed of an essential catalytic core enzyme and one of several alternative sigma (σ) factors [50]. *C. thermocellum* contains a single copy of the *rpoB* gene and the PM strain has a non-synonymous SNP mutation (E885K) in a strongly conserved amino acid based on the Clustalw alignment (File S4). This mutation potentially changes the specificity, activity and/or stability of the RNA polymerase. Since, the RNA polymerase contacts every promoter in the genome; a single amino acid substitution in the critical portions of the enzyme may lead to global changes in gene expression and cellular function [50]. By comparison, Alper et al. (2007) randomly mutated *rpoD*, which encodes the main sigma factor in *Escherichia coli* [51]. Mutations to the sigma factor were able to elicit changes in phenotype which confer tolerance to ethanol, metabolite overproduction, and multiple, simultaneous phenotypes [51]. The *rpsL* gene (Cthe_2727) produces the ribosomal protein S12 [52] which is part of the 30S ribosomal subunit. Alterations in *rpsL* are known to confer resistance to the error-inducing antibiotic streptomycin [53]. The *rpsL* mutation (A14V) occurs in a poorly conserved location based on the Clustalw alignment (File S5). Together, mutations in the subunits of RNA polymerase and ribosomes have significant potential to change the overall protein expression profile in the cell. However, these mutations do not significantly change the gene expression level of the *rpoB* and *rpsL* genes in any of the test conditions (data not shown). In addition, mutations in both *rpoB* and *rpsL* have been shown to impart increased tolerance to solvents and aromatic compounds, such as 4-hydroxybenzoate, by blocking their uptake through the membrane transport system [52]. Therefore, it is possible that these mutations contribute to the enhanced hydrolysate tolerance of the PM. 

RNase G (Cthe_0158) endoribonuclease is required for the normal decay of several transcripts including mRNA from two genes in the glycolysis pathway, enolase (eno) and acetaldehyde dehydrogenase (*adhE*) [54]. When the *rng* protein is disrupted, the *eno* and *adhE* proteins are known to be over produced as a consequence of mRNA stabilization and the resulting increase of their half-lives [54]. Although the mutation does not occur in a known domain, it is possible that the deletion of codons 438 through 486 of 498 may disrupt the functionality of the *rng* protein and decrease the rate of RNA degradation in the PM. A reduction in mRNA degradation can either increase the activity of mRNA or maintain mRNA activity with reduced transcriptional effort. The *rng* gene is significantly upregulated in the WT in the presence of hydrolysate (Figure 4) which would result in more rapid mRNA turnover. Upregulation of *rng* does not occur in PM, which, when combined with the potential disruption of *rng* functionality would result in significant reduction of mRNA turnover. Consistent with the differences in *rng* activity, the activity of *adhE* (Cthe_0423) and *eno* (Cthe_0143) transcripts is decreased in the WT in hydrolysate medium whereas the PM maintains constant activity (See Figure 4). Since DNA is particularly sensitive to damage during transcription, reduced mRNA turnover rates would also lead to reduced DNA damage by the hydrolysate, and could conserve energy and increase metabolic efficiency in stable environments that do not require frequent changes in gene expression profile. Furthermore, constant expression of the *eno* and *adhE* genes in the PM may allow the organism to maintain flux through the acetyl CoA to ethanol pathway. Since this pathway is a key mechanism by which NAD^+^ is recycled, maintenance of *adhE* activity may contribute to greater flux through the glycolysis pathway. Together, the benefits of reduced mRNA turnover may play a significant role in the increased tolerance and faster growth of the PM. 

### Altered Energy Metabolism

The faster growth rate of the PM and increased product yields suggest that mutations in the PM result in an altered metabolism. Furfural and HMF are reduced to their corresponding alcohols through NAD(P)H dependent reactions, thereby reducing the need to recover NAD^+^ through ethanol formation [48,55]. In the WT, the Cthe_0422-3 operon, which encodes for the Rex (redox) repressor and *adhE*, is down regulated in the presence of hydrolysate (Figure 4). Surprisingly, WT ethanol yields increases in hydrolysate medium despite down regulation of *adhE*. In *Clostridium acetobutylicum*, Rex regulates the expression of solventogentic genes, including *adhE2*, in response to the NAD^+^/NADH ratio. The PM had a mutation in the non-coding region upstream of the Cthe_0422-3 operon which seems to maintain its expression level in hydrolysate medium (Figure 4). While it is unclear whether *adhE* activity in the WT is down regulated by Rex or by another mechanism, such as the activity of RNase G discussed above, the end result is that the PM maintains greater activity of *adhE* in the presence of hydrolysate, contributing both to greater ethanol yields and availability of NAD^+^ for upstream glycolytic reaction steps. As to be expected with the increased expression of *adhE* compared to the WT, the PM has a greater ethanol to acetic acid ratio (0.43:1) when compared to the WT strain (0.35:1) (p-value = 1.46E-05) in standard medium. This mutation might both enhance the growth of the PM in standard medium and impart greater tolerance to the hydrolysate media. 

The PM has a mutation in the non-coding region 127 bp upstream of the first gene in the operon (Cthe_2602-09) coding for the ATPase complex. The PM has significantly increased expression levels for the entire ATPase operon compared to the WT in all conditions tested (Figure 4). Of the single colony isolates, Isolates 1-6 also have a mutation in Cthe_2603 (I4M) which encodes for the C subunit of the ATP synthase (Table S1); however, the effect of this mutation is unknown at this time. 

The ATPase obtains energy from the transmembrane transport of ions (H^+^/Na^+^) along the electrochemical gradient to generate ATP from the substrates adenosine diphosphate (ADP) and inorganic phosphate (P_i_). An increase in the activity of ATPase complex can provide additional energy for biosynthetic processes. It has been proposed that ATPase might also work in conjunction with the membrane bound Ferredoxin-dependent Ech-type NiFe-hydrogenase (Cthe3013-3024) for the production of molecular hydrogen to dispose of excess reducing equivalents generated during carbohydrate catabolism and to create the necessary H^+^/Na^+^ gradients to power ATP synthesis [27]. However, the statistically similar rates of H_2_ production per glucan utilization of the WT and PM in both 0 and 10% v/v *Populus* hydrolysate media does not support this argument (Figure S6A and S6C). Alternatively, ATPase may operate against the H^+^ gradient at the expense of ATP [6]. In this case ATPase may act to increase the intercellular pH thereby providing a tolerance mechanism for undissociated forms of weak acids in the hydrolysate. Given the conflicting evidence, it is not possible at this time to ascertain the role of the up regulation of ATPase with certainty. 

Cells respond to the effect of inhibitory compounds by alterations in the biosynthetic programs to produce metabolites, such as amino acids, that counter toxic effects [6]. The PM contained several mutations involved in amino acid production which are similar to those seen by an ethanol tolerant mutant under similar conditions [3] and may enhance the tolerance of the PM to hydrolysate as well. The PM has two mutations involved with glutamate catabolism. A mutation to a gene in the arginine pathway, *argD* (Cthe_1866 E55G) resulted in a pfam score of +1180. This common mutation has been previously observed in the *C. thermocellum* DSM 1313 strain [3]. Another mutation to a gene in the proline pathway, *proB* (Cthe_1766 A149T) resulted in a pfam score of -18 which suggests similar functionality. This mutation is found in *Clostridium phytofermentans*. These two mutations could suggest a shift from proline production to arginine production in PM which seems to be a beneficial mutation that commonly occurs (see Figure 5B). An increase in amino acid production can also help overcome weak acid stress. The decarboxylation and antiport of the amino acids glutamate and arginine generates a net efflux of intracellular protons thus increasing intracellular pH [6,56] thereby suggesting a possible mechanism by which proton gradients for ATP synthesis may be maintained. 

There are two copies (Cthe_1569 and Cthe_1842) of the *metY* gene in *C. thermocellum*, which produces L-cysteine, both of which have mutations in the PM that seem to inactivate the proteins (Figure 5C). Cthe_1569 has a stop sequence inserted at codon 229 and the non-synonymous SNP in a highly conserved region of Cthe_1842 (P29Q) results in a pfam score of -1198. These two mutations essentially cut off the conversion of serine and hydrogen sulfide to L-cysteine. Miller et al. (2009) showed that supplementing fermentation media with cysteine improved furfural resistance the greatest out of the five amino acids tested [57]. Since MTC media contains L-cysteine the benefits of forgoing its synthesis may have provided selective pressure favorable to these mutations [57].

The *spo0A* transcription factor is responsible for the initiation of sporulation [58,59]. *Spo0A* has been shown to repress transcription of the transcriptional pleiotropic regulator of transition state genes (*abrB*) and to activate operons encoding stage II sporulation genes [58,60]. The *abrB* product acts to minimize the expression of extraneous genes that interfere with the maximal rate of growth of the cell under nutrient-excess conditions [59]. *Spo0A* mutants are unable to go into sporulation and are thought to be locked in exponential growth since they continue to grow under nutritional conditions that would normally induce sporulation. Essentially they appear to maintain growth until the nutrients are exhausted, whereupon cell lysis occurs [59]. This behavior is consistent with the PM growth kinetics and other *spo0A* defective mutants [60-62]. There are two copies of *spo0A* in *C. thermocellum*, Cthe_0812 and a distantly related homologue Cthe_3087. The PM has a stop codon placed early in the coding region of Cthe_3087 which should disrupt the protein function. As would be expected, the gene expression of Cthe_3087 is not significantly different for the PM in any test condition. Cthe_0812 is significantly downregulated by an unknown mechanism for the PM under all test conditions (Figure 4). The disrupted expression of both *spo0A* proteins in the PM results in increased expression of *abrB* (Cthe_2100) in standard medium (Figure 4). The expression of *abrB* is down regulated in the presence of hydrolysate in both the WT and PM strains, consistent with the decrease in growth of the WT in 10% v/v *Populus* hydrolysate medium and the PM in 17.5% v/v *Populus* hydrolysate medium. 

### Genes and mutations with unknown function

Of the three mutations in non-coding regions of genes with unknown function, the first mutation (located at position 2179038) is an insertion of a string of nine adenine nucleotides 388 bp upstream of Cthe_1836, a hypothetical protein that results in an increase in its expression in the PM compared to the WT. The second mutation (located at position 3151358) is an INDEL of unknown size and 245 bp downstream of the 3’ end of Cthe_2670, a hypothetical protein and 221 bp upstream of the 3’ end of Cthe_2671, mutator type transposase. This mutation seems to be related to an increase in expression of both genes in the PM in the presence of hydrolysate. The third mutation (located at position 2732545) is unique to PM Isolate 6 and is a deletion of 67 bp in an intergenic region (Table S1) more than 3000 bp from the closest gene. Three additional mutations occurred in the coding regions of genes of unknown function. Cthe_0531 was mutated at the C30Y position; however, a BLAST search returned no similar genes with known function. The BLAST search of Cthe_0291, a synonymous SNP at codon 19, returned a number of genes annotated as the thioredoxin, *yyaL*, gene which are responsible for maintaining a cellular reducing environment and fulfill an important role in DNA synthesis and protein repair [63]. Even if Cthe_0291 is a thioredoxin homologue, the synonymous nature of the SNP decreases the likelihood that this mutation contributes to the tolerant phenotype. The third mutation is unique to Isolate 6 and is an INDEL in the hypothetical protein Cthe_1480. The BLAST search returned a number of genes annotated as RND family transporter and *mmpL* domain-containing proteins. The pfam score of -492 suggest that this mutation is a loss of function. Although there are differences in the gene expression levels for these genes (Figure 4), the potential impacts of these mutations are unknown at this time.

### Extraneous or detrimental mutations

The PM has three mutations affecting genes associated with the cellulosome. These mutation may result in the conservation of cell resources when grown on soluble hydrolysate, but could be detrimental to cell growth on solid biomass. Cthe_2119 (*RsgI6*), is involved in regulating the expression of cellulosomal genes in the presence of xylans and cellulose [20,23,64], contains a synonymous SNP in codon 415. Since the mutation is outside of the catalytic domain and does not result in a change in expression, it is unlikely to cause a significant change in phenotype. A mutation (E458K) in *bglX* (Cthe_1256) results in a pfam score of -539 suggesting a possible loss of function. The gene encodes for a GH3 family *beta-D-glucoside glucohydrolase* which breaks the β-1,4 glucosidic bonds of cellobiose to produce glucose monomers [65]. Hydrolysate seemed to induce expression of this gene in the WT, but had no effect on the PM. Therefore, the mutation seems to negatively affect both the functionality and the expression level of the GH3 protein PM Isolate 6 also has a unique mutation in cellulosome anchoring protein Cthe_3078. The mutation is a synonymous SNP in codon 1013, which is outside of a known domain. In the WT the expression of this gene increases in the presence of hydrolysate. The expression in the PM is repressed in 0% v/v *Populus* hydrolysate and is insensitive to hydrolysate concentration. 

The PM also had mutations related to the ATP-binding cassette (ABC) and Major Facilitator Superfamily 1 (MFS_1) sugar transporter systems. The Cthe_1018-1020 ABC transport system has been suggested to play a major role in cellodextrin assimilation [10]. A mutation (A99V) in the extracellular binding protein of the complex, Cthe_1020 (*cbpB*) is associated with a pfam score of -277. Although, the role of this mutation is not clear, the negative pfam suggests that it might be detrimental to the function of the protein. Solutes transported by the MFS_1 family include galactose, arabinose, xylose, and glucose in bacteria [66]. The PM has two mutations in the MFS_1 gene (Cthe_1202); one is in the non-coding region 82 bp upstream of the 5’ end and the other is R343G. There is no significant change in gene expression between the WT and PM (data not shown) so it is unlikely that the mutation in the non-coding region affects gene expression; however, the mutation in the coding region may change the selectivity or specificity of this protein. Since the PM was evolved only on soluble cellobiose, it is possible that these mutations are a result of an attempt to conserve energy and may be detrimental to the cell when grown on more complex substrates.

The PM also has two mutations along the pyrimidine pathway (Cthe_0947-0953). The gene *carB* (Cthe_0949 P361H) encodes for a subunit of the carbamoyl-P synthase enzyme which catalyzes its synthesis from ammonia and ATP [2]. The other mutation in *pyrDII* (Cthe_0948 D173H) is located on the pathway from carbamoyl-P to orotate. These two mutations do not result in a significant change in the pfam score or gene expression (data not shown); therefore, these mutations are unlikely to contribute significantly to the tolerant phenotype (see Figure 5B).

PM Isolate 6 also has two extraneous unique mutations. The first mutation (K559R) occurs in a gene that encodes a multi-sensor signal transduction histidine kinase (Cthe_1393). The pfam score of 55 suggest that this mutation does not greatly affect the functionality of the protein. There is also no significant change in expression of this gene. The other mutation is in the urea ABC transporter ATP-binding protein *urtE* (Cthe_1819). The mutation (N99I) is outside of a known domain and does not result in a significant change in gene expression. 

Future work includes a global analysis of gene expression and kinetic modeling of growth for the PM and WT strains to further understanding the mechanisms of *Populus* hydrolysate tolerance. Additional analysis of these mutations could be pursued using system biology methods, genetic tools, biochemical assays and strain engineering.

##  Conclusion

This study is the first investigation of the mechanism of tolerance for *C. thermocellum* to inhibition by a complex hydrolysate generated during pretreatment, in this case using *Populus* hydrolysate. Genome sequencing used in the study proved to be a useful tool to reveal cellular evolution to stress such as that provided by the composition of *Populus* hydrolysate. Analysis of the mutations included a longitudinal analysis, a pan genomic analysis, and a hotspot analysis. Additional systems biology tools including pfam scores and RNA_seq analysis led to the identification of the multiple mutations most likely to confer tolerance to the *Populus* hydrolysate based of change in function and gene expression. These mutations are located in several simultaneous mechanisms of action, including increases in cellular repair, and altered energy metabolism. This study may provide the building blocks for the construction of an industrially robust organism for economical ethanol production. 

## Supporting Information

Figure S1 **Growth comparison of WT and 7 different single colony isolates in standard MTC media and 17.5% v/v *Populus* Hydrolysate.** The OD was referenced to an uninoculated sample of medium incubated with the samples. The PM and WT have the ability to degrade two of the compounds in the *Populus* hydrolysate which lightens the color and leads to the negative OD.
(TIF)

Figure S2 **PM and WT growth comparison on insoluable substrate (Avicel) as a function of pellete and supernatant nitrogen concentration taken over time.**
(TIF)

Figure S3 **Longitudinal Genomic Analysis in which six populations samples along the evolutionary process were sequenced to determine when the mutations occurred.** (A) Daily transfers over the entire evolutionary process in various hydrolysate concentrations. The arrows indicate where in the process the different population samples were taken for sequencing. Samples were considered either final mutations if taken at the end of a given concentrations evolutionary process or intermediate mutants if taken during the middle of the processes. (B) The total number of mutations that occurred for each population by catergory of the pan genomic analysis.
(TIF)

Figure S4 **Pan Genomic Analysis of the seven single colony isolates from the final 17.5% v/v *Populus* hydrolysate-tolerant mutant strain.** The core mutations are most likely responsible for the increased robustness since they are shared by all isolates.
(TIF)

Figure S5 **Distribution analysis of mutations data.** Syn: synonymous SNP, Non-Syn: Non-Synonymous SNP, INDEL: insertion or deletion, and STOP: stop codon inserted.
(TIF)

Figure S6 **In Comparison of the g/L Dry Cell Weight (DCW) versus g/L H_2_ production in A) 0% v/v *Populus* hydrolysate and B) 10% v/v *Populus* hydrolysate.** Compaison of the g/L DCW versus g/L CO_2_ production in C) 0% v/v *Populus* hysrolysate and D) 10% v/v *Populus* hydrolysate. The comparisons are for the PM and WT strains for the exponential growth phase using a sacraficial test tube experiment. Trendlines were added to show the rate of production.
(TIF)

File S1 ***Clostridium thermocellum* genomic analysis** : An excel File containing the full list of possible mutations received from JGI.
(XLSX)

File S2 **Differential Gene Expression Profiles** : An excel file containing the significantly differentially expressed genes based of the ANOVA results.
(XLSX)

File S3 **Cthe_2376-alignment: A text containing the Clustawl alignment of the SNP in Cthe_2376 with the top 100 hits.**
(TXT)

File S4 **Cthe_2724-alignment: A text containing the Clustawl alignment of the SNP in Cthe_2724 with the top 100 hits.**
(TXT)

File S5 **Cthe_2727-alignment: A text containing the Clustawl alignment of the SNP in Cthe_2727 with the top 100 hits.**
(TXT)

Table S1 **Selected mutations for verification of mutations in the WT and PM Isolate 6.** The putative mutations included for PCR verification 19 high confidence mutations and 9 false postive mutations. The table lists the gene name, position of the mutation, type of mutations as determined by JGI (Syn: synonymous SNP, Non-Syn: Non-Synonymous SNP, NC: non-coding region, STOP: stop codon inserted, INDEL: insertion or deletion, FP- false positive, ISE – instrument specific error or ???- unknown), the identity of the base in question determined in the PCR products of the WT and PM strains (WT:PM), and the final determination of whether a real mutation or a false positive mutation occurred. Both the forward and reverse strands were sequenced for each mutation listed.
(PDF)

Table S2 **Shared and uniquie mutations in the seven single colony isolates.** ‘X’ marks which isolate contained the mutation. * Indicates that the mutation was detected in the final 17.5% v/v *Populus* hydrolysate tolerant mutant populuation sample at less than 2%. The rest of the mutations were not detected in the population sample. None of the mutations were detected in earlier population samples. Type of mutations: Syn: synonymous SNP, Non-Syn: Non-Synonymous SNP, NC: non-coding region, STOP: stop codon inserted, INDEL: insertion or deletion.
(PDF)

Table S3 **P-values determined by Analysis of Covariance to determine statistically significant differences in growth parameters.** The growth rates and yields are calculated from slopes. For growth rate the slope is calculated from a plot of ln(biomass) vs. time. For yields, the slope is calculated from product vs. substrate. Statistical comparisons of slopes from different experiments were conducted using Analysis of Covariance. Briefly, a multiple regression was conducted for each pair-wise comparison for the dependent variable y as a function of the independent variables x and z, where z encodes the treatment (0 for treatment A and 1 for treatment B). The reported p-values represent the statistical significance of the treatment variable term in the multiple regression. P-values < 0.05 is considered statistically significant.
(PDF)
