# Supplementary figures and images for: Industrial Robustness: Understanding the Mechanism of Tolerance for the Populus Hydrolysate-Tolerant Mutant Strain of Clostridium thermocellum
Source: PLoS One. 2013 Oct 21;8(10):e78829. doi: 10.1371/journal.pone.0078829 (PMC3804516; doi:10.1371/journal.pone.0078829)

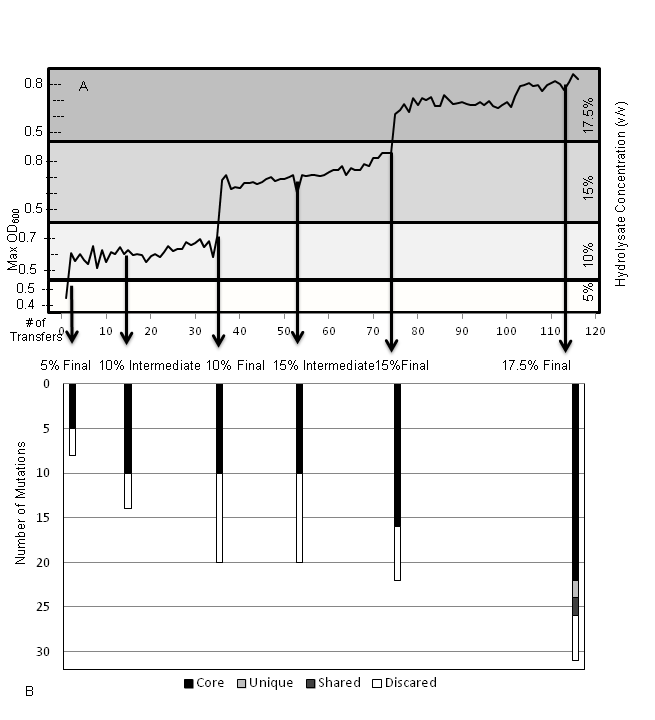

Supplement: Figure S3 — Longitudinal Genomic Analysis in which six populations samples along the evolutionary process were sequenced to determine when the mutations occurred. (A) Daily transfers over the entire evolutionary process in various hydrolysate concentrations. The arrows indicate where in the process the different population samples were taken for sequencing. Samples were considered either final mutations if taken at the end of a given concentrations evolutionary process or intermediate mutants if taken during the middle of the processes. (B) The total number of mutations that occurred for each population by catergory of the pan genomic analysis. (TIF) [file pone.0078829.s003.tif]
