# Supplementary material for: Industrial Robustness: Understanding the Mechanism of Tolerance for the Populus Hydrolysate-Tolerant Mutant Strain of Clostridium thermocellum
Source: PLoS One. 2013 Oct 21;8(10):e78829. doi: 10.1371/journal.pone.0078829 (PMC3804516; doi:10.1371/journal.pone.0078829)
Supplement: Table S1 — Selected mutations for verification of mutations in the WT and PM Isolate 6. The putative mutations included for PCR verification 19 high confidence mutations and 9 false postive mutations. The table lists the gene name, position of the mutation, type of mutations as determined by JGI (Syn: synonymous SNP, Non-Syn: Non-Synonymous SNP, NC: non-coding region, STOP: stop codon inserted, INDEL: insertion or deletion, FP- false positive, ISE – instrument specific error or ???- unknown), the identity of the base in question determined in the PCR products of the WT and PM strains (WT:PM), and the final determination of whether a real mutation or a false positive mutation occurred. Both the forward and reverse strands were sequenced for each mutation listed. (PDF) [file pone.0078829.s012.pdf]

Gene Name	Position	JGI Mutation Type	WT:PM	Results
Cthe_0158	199088-199233	DEL	Del	Real
upstream Cthe_0422	530675	NC	T:G	Real
Cthe_0766	931066	ISE	A:A	False positive
Cthe_0948	1135183	Non-Syn	C:A	Real
Cthe_0949	1137862	Non-Syn	C:A	Real
Cthe_1020	1221346	Non-Syn	C:T	Real
Cthe_1256	1523834	Non-Syn	G:A	Real
Cthe_1393	1702173	Non-Syn	A:G	Real
Cthe_1444	1762902	ISE	A:A	False positive
Cthe_1528	1854473	ISE	G:G	False positive
Cthe_1569	1897805	STOP	G:T	Real
Cthe_1604	1935519	ISE	T:T	False positive
Cthe_1766	2088723	Non-Syn	G:A	Real
Cthe_1819	2153823	Non-Syn	A:T	Real
upstream Cthe_1831	2171049	ISE	G:G	False positive
Cthe_1842	2187817	Non-Syn	C:A	Real
Cthe_1866	2212180	Non-Syn	A:G	Real
Cthe_2018	2397410	???	T:T	False positive
Cthe_2091	2486924	ISE	A:A	False positive
Cthe_2119	2517570	Syn	G:A	Real
Cthe_2376	2838931	Non-Syn	G:T	Real
Cthe_2418	2888569	ISE	A:A	False positive
Upstream Cthe_2602	3073025	NC	C:T	Real
Cthe_2603	3073931	Non-Syn	A:G	Real
Cthe_2724	3214087	Non-Syn	G:A	Real
Cthe_2727	3219211	Non-Syn	C:T	Real
Cthe_2985	3503182	ISE	A:A	False positive
Cthe_3087	3642539	STOP	C:T	Real
