# Supplementary material for: Industrial Robustness: Understanding the Mechanism of Tolerance for the Populus Hydrolysate-Tolerant Mutant Strain of Clostridium thermocellum
Source: PLoS One. 2013 Oct 21;8(10):e78829. doi: 10.1371/journal.pone.0078829 (PMC3804516; doi:10.1371/journal.pone.0078829)
Supplement: Table S2 — Shared and uniquie mutations in the seven single colony isolates. ‘X’ marks which isolate contained the mutation. * Indicates that the mutation was detected in the final 17.5% v/v Populus hydrolysate tolerant mutant populuation sample at less than 2%. The rest of the mutations were not detected in the population sample. None of the mutations were detected in earlier population samples. Type of mutations: Syn: synonymous SNP, Non-Syn: Non-Synonymous SNP, NC: non-coding region, STOP: stop codon inserted, INDEL: insertion or deletion. (PDF) [file pone.0078829.s013.pdf]

Isolate 1	Isolate 2	Isolate 3	Isolate 4	Isolate 5	Isolate 6	Isolate 7	Position	Name	Product	Type of Mutation	Placement	Strand
	X						212735	Cthe_0175	polysaccharide deacetylase	Non-Syn	S280G	+
	X						606073	Cthe_0492	CheC, ingibitor of MCP methylation	Non-Syn	E53K	+
						X	662249	Cthe_0541	peptidase M24	Syn	N286N	+
X			X				1187137	Cthe_0992	ribosomal protein L7Ae/L30e/S12e/Gadd45	Syn	T95T	-
			X				1221367	Cthe_1020	extracellular solute-binding protein family 1	Non-Syn	A92V	-
X							1230221	Cthe_1028	acetate kinase	INDEL		
X			X				1342025	Cthe_1127*	integrase family protein	Non-Syn	K5E	+
						X	1663282	NC	115 bp 5' end of Cthe_1368: S-layer domain-containing protein	INDEL		
					X		1702173	Cthe_1393	multi-sensor signal transduction histidine kinase	Non-Syn	K559R	-
				X			1717600	NC	1735 bp from 5' end of Cthe_1401	INDEL	Deletion of 1717552-1717617	
					X		1799942	Cthe_1480	hypothetical protein	INDEL		
						X	1880654	NC	606 bp from end of F Cthe_1550 and 868 bp from end of R Cthe_1552	NC		NC
					X		2153823	Cthe_1819	urea ABC transporter, ATP-binding protein UrtE	Non-Syn	N99I	-
						X	2270432	Cthe_1909	copper-amine oxidase-like domain-containng protein	Syn	V423V	-
				X			2421066	NC*	1519 bp from 5' end of Cthe_2036	NC		NC
				X			2614779	Cthe_2193	carbohydrate binding family 6	Non-Syn	I601V	-
				X			2699028	Cthe_2270	ABC transporter related protein	Syn	A535A	-
					X		2732545	NC	3524 bp from 3' end of Cthe_2295 and 8703 bp from 3' end of Cthe_2297	INDEL	Deletion of 2732545-2732612	
						X	2997777	NC	98 bp from 5' end of Cthe_2529: delta-aminolevulinic aicd dehydratase	DEL		
X	X	X	X	X	X		3073931	Cthe_2603*	ATP synthase F0, C subunit (operon), signal peptide, amino acid has several substitutions	Non-Syn	I4M	+
						X	3073969	Cthe_2603	ATP synthase F0, C subunit	Non-Syn	V17A	+
	X						3086626	Cthe_2611	S-layer domain-containing protein	Non-Syn	M1473T	+
X			X				3136682	NC	167 bp from 3' end of Cthe_2655: AbrB family transcriptional regulator	INDEL	Insertion: TTTTTTT	
						X	3224162	Cthe_2731	RNA polymerase, sigma-24 subunit, ECF subfamily	Non-Syn	R42G	+
				X			3233583	Cthe_2741	ATP-dependent Clp protease, ATP-binding subunit Clpx	Non-Syn	E20G	+
				X			3525977	Cthe_3003*	hydrogenase, Fe-only	Non-Syn	V329I	-
					X		3628267	Cthe_3078	cellulosome anchoring protein cohesin region	Syn	V1013V	+
