# Supplementary material for: Industrial Robustness: Understanding the Mechanism of Tolerance for the Populus Hydrolysate-Tolerant Mutant Strain of Clostridium thermocellum
Source: PLoS One. 2013 Oct 21;8(10):e78829. doi: 10.1371/journal.pone.0078829 (PMC3804516; doi:10.1371/journal.pone.0078829)
Supplement: Table S3 — P-values determined by Analysis of Covariance to determine statistically significant differences in growth parameters. The growth rates and yields are calculated from slopes. For growth rate the slope is calculated from a plot of ln(biomass) vs. time. For yields, the slope is calculated from product vs. substrate. Statistical comparisons of slopes from different experiments were conducted using Analysis of Covariance. Briefly, a multiple regression was conducted for each pair-wise comparison for the dependent variable y as a function of the independent variables x and z, where z encodes the treatment (0 for treatment A and 1 for treatment B). The reported p-values represent the statistical significance of the treatment variable term in the multiple regression. P-values < 0.05 is considered statistically significant. (PDF) [file pone.0078829.s014.pdf]

p-value of regression analysis					
	PM vs. WT		0% vs. 10/17.5% v/v hydrolysate		
	0% v/v hydrolysate	10% v/v hydrolysate	PM 0 vs. 10%	PM 0 vs. 17.5%	WT 0 vs. 10%
Growth Rate	6.35E-13	3.71E-23	0.8689	0.0175	1.44E-09
Cell Yield	3.37E-05	1.44E-10	0.0247	0.0066	0.1116
Ethanol Yield	0.0337	9.04E-05	0.0002	1.35E-05	1.25E-08
Acetic Acid Yield	0.8622	0.1210	0.0001	9.72E-07	0.0700
Hydrogen Yield	0.295	0.314			
Carbon Dioxide Yield	0.028	0.180			

P-values for comparison of growth rates between Q-plus fermenters and Balch tubes: PM in 0% hydrolysate (0.133), PM in 10% hydrolysate (0.659), WT in 0% hydrolysate (0.597), and WT in 10% hydrolysate (0.201).
